# The CorInnova Implantable Cardiac Assist System for Direct Cardiac Compression

**DOI:** 10.31083/j.rcm2306211

**Published:** 2022-06-09

**Authors:** George V. Letsou, Christina M. Bolch, Erica C. Hord, William C. Altman, Boris Leschinsky, John C. Criscione

**Affiliations:** ^1^Baylor St. Luke’s Medical Center, Houston, TX 77030, USA; ^2^CorInnova, Inc., Houston, TX 77021, USA; ^3^Department of Biomedical Engineering, Texas A&M University, College Station, TX 77843, USA

**Keywords:** heart-assist devices, assisted circulation, cardiopulmonary resuscitation, cardiac output, stroke volume, minimally invasive surgical procedures

## Abstract

The CorInnova cardiac compression device (CorInnova, Inc., Houston, TX, USA) is designed to provide direct biventricular support, increase cardiac output, and improve ventricular unloading in patients with heart failure. Placed within the pericardium and surrounding both ventricles, the device has two concentric sets of thin-film polyurethane chambers: (1) inner (epicardial) saline-filled chambers that conform intimately to the epicardial surface, eradicating any gaps in the interface between the device and the heart; and (2) outer air-filled chambers cycled to provide epicardial compression during systole and negative epicardial pressure during diastole, consistent with physiological cardiac contraction and relaxation. A superelastic, collapsible Nitinol frame gives the device structure, enables minimally invasive self-deployment, and enhances diastolic filling. Preclinical testing has been extremely promising, with improvements in cardiac output and other cardiac parameters in animal heart failure models. This potentially transformative technology is moving rapidly toward first-in-human use. The CorInnova device may provide an effective device-based solution for patients with heart failure who currently have few or limited mechanical cardiac support options, including patients with biventricular cardiac failure, those with right heart failure, those who are older, and those who are of smaller size. It can be removed easily and requires minimal maintenance. An important, unique feature of this technology is that it provides mechanical cardiac assistance without blood contact or need for anticoagulation. The CorInnova device may be particularly important for those patients who have contraindications to anticoagulation due to allergy, neurological bleeds, or preexisting hemorrhage. No other mechanical circulatory support device addresses these underserved heart-failure populations.

## 1. Introduction

Heart failure affects 5–6 million patients each year in the United States, with 500,000 to 1 million new diagnoses each year [1, 2, 3]. It occurs in approximately 1% of the US population older than 40 years, and its incidence increases with age [4]. The United States spends $31 billion annually on care related to heart failure, more than for any other diagnosis-related group [5]. Costs for the heart failure diagnosis-related group are estimated to be $70 billion by 2030 [5]. Current heart failure therapies include medical regimens, surgical procedures such as coronary artery bypass surgery and valvular heart surgery, and mechanical cardiac assistance [6].

Mechanical cardiac assistance plays a rather limited role in the treatment of heart failure, given that all currently available devices require invasive intravascular placement. Mechanical cardiac support is difficult or contraindicated in various clinical scenarios. For example, diabetes and peripheral arterial disease are comorbidities in as many as 30% of patients with heart failure [7, 8, 9, 10], bleeding, coagulopathy, and difficult peripheral access due to small patient size are common in these patients [11], and being older than 70 years of age is often a relative contraindication to mechanical cardiac assistance [11]. Although women are diagnosed with heart failure as frequently as men, 75% of ventricular assist devices are placed in men. This is often attributed to size differences between men and women (but other explanations, such as the higher prevalence of heart failure with preserved ejection fraction in women, may also be important) [12]. Diastolic or biventricular mechanical cardiac assistance is difficult to provide with current techniques, and there is need for a less-invasive, non–blood-contacting device. Despite these difficulties, approximately 150,000 mechanical cardiac assist devices are placed each year in the United States, including 100,000 intra-aortic balloon pumps (IABPs), 25,000 percutaneous catheter-based heart pumps, and 3000 left ventricular assist devices [13].

**Fig. 1.**
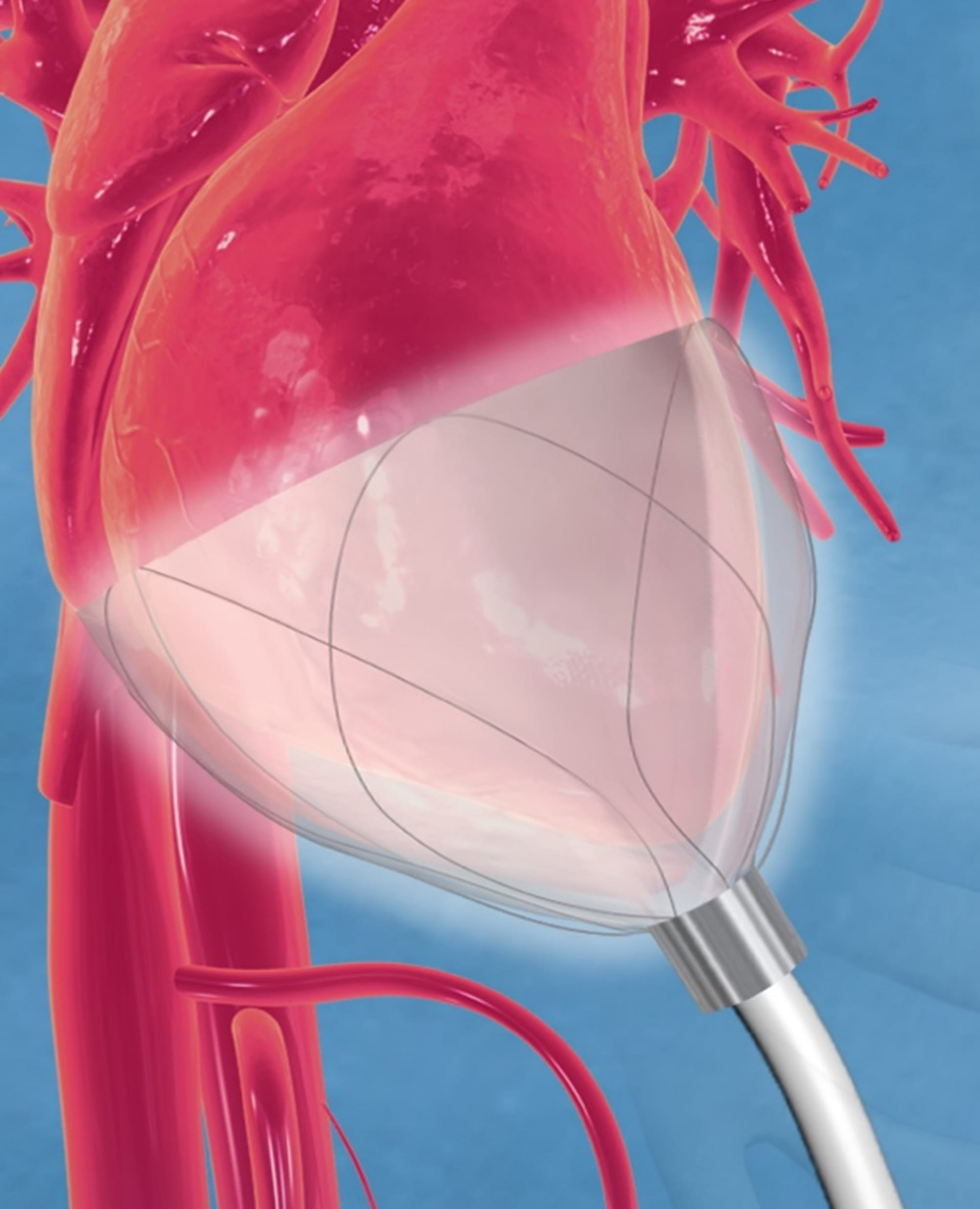
**CorInnova device in place and encircling both left and right ventricles**. The Nitinol frame supports inner saline-filled chambers and outer air-filled chambers that inflate and deflate cyclically. The driveline extends outwards from the device’s apex. Image courtesy of CorInnova, Inc.

To addresses these barriers, CorInnova, Inc. (Houston, TX) has developed an implantable cardiac compression and relaxation device (Fig. 1) that is applied to the heart’s external surface. As the only mechanical cardiac assist device that does not require intravascular placement, the CorInnova device has no blood contact and therefore does not require anticoagulation. It is effective in patients of all sizes, including smaller patients in whom other devices may be difficult to implant. Age is not a relative contraindication. The CorInnova device provides both systolic and diastolic cardiac assistance. Because the device is implanted through a small thoracotomy (and potentially can be implanted by using endoscopic techniques in the catheterization laboratory), poor peripheral arterial access is not a contraindication. The CorInnova device is less invasive than presently available ventricular assist devices, can be manufactured to fit smaller individuals, and should address the large population with heart failure who may not be ill enough for left ventricular assist device placement.

This article reviews the physiological basis of mechanical cardiac compression for hemodynamic support, several of the devices that have been designed to provide such mechanical support, and specifics concerning the novel CorInnova device.

## 2. Cardiac Compression and Resuscitation: A Brief History

Initial efforts at cardiopulmonary resuscitation began 500 years ago. Respiratory bellows were used for resuscitation beginning in the 1500s. Mouth-to-mouth resuscitation was initially described in 1732 by the Scottish physician William Tossach [14]. In 1775, the Danish veterinarian Abildgaard discovered that electrical countershocks could restore heart rhythm after cardiac arrest in chickens [15, 16]. Eighty years later, London physicians Marshall Hall and Henry Sylvester achieved resuscitation by physically repositioning the patient’s head from face-up to sideways and by using chest pressure and arm lifts to compress and expand the thorax, respectively [17, 18]. Working in Florence, Italy, Moritz Schiff described the return of circulation in response to open cardiac massage in 1874 [19]. Open cardiac massage remained the standard of care for most of the first half of the 20th century.

In 1878, Germany’s Rudolph Boehm demonstrated that external cardiac compression could restore circulation in cats [20], and by 1903 George Crile in Cleveland, Ohio had shown that external chest compression could restore canine circulation [21]. Dr Crile went on to demonstrate successful closed-chest cardiac compression in a case of human resuscitation, but the technique did not gain acceptance [20]. Glenn and colleagues at Yale described 42 cases of attempted cardiac resuscitation using closed-chest techniques in 1954 [22]. It was not until 1960, after further advances in rescue ventilation and cardiac defibrillation, that Kouwenhoven, Safar, and Jude introduced the concept of cardiopulmonary resuscitation, which combined mouth-to-mouth breathing with external closed-chest cardiac compression [23]. Ever since, closed-chest compression with mask ventilation has been the mainstay for resuscitation after out-of-hospital and in-hospital cardiopulmonary arrest.

Although the effectiveness of external manual chest compression and of direct manual cardiac compression was well established by the 1960s [24, 25], successful hemodynamic support with direct cardiac compression devices has been much harder to achieve. Several devices designed for direct cardiac compression are described in the following paragraphs.

**Fig. 2.**
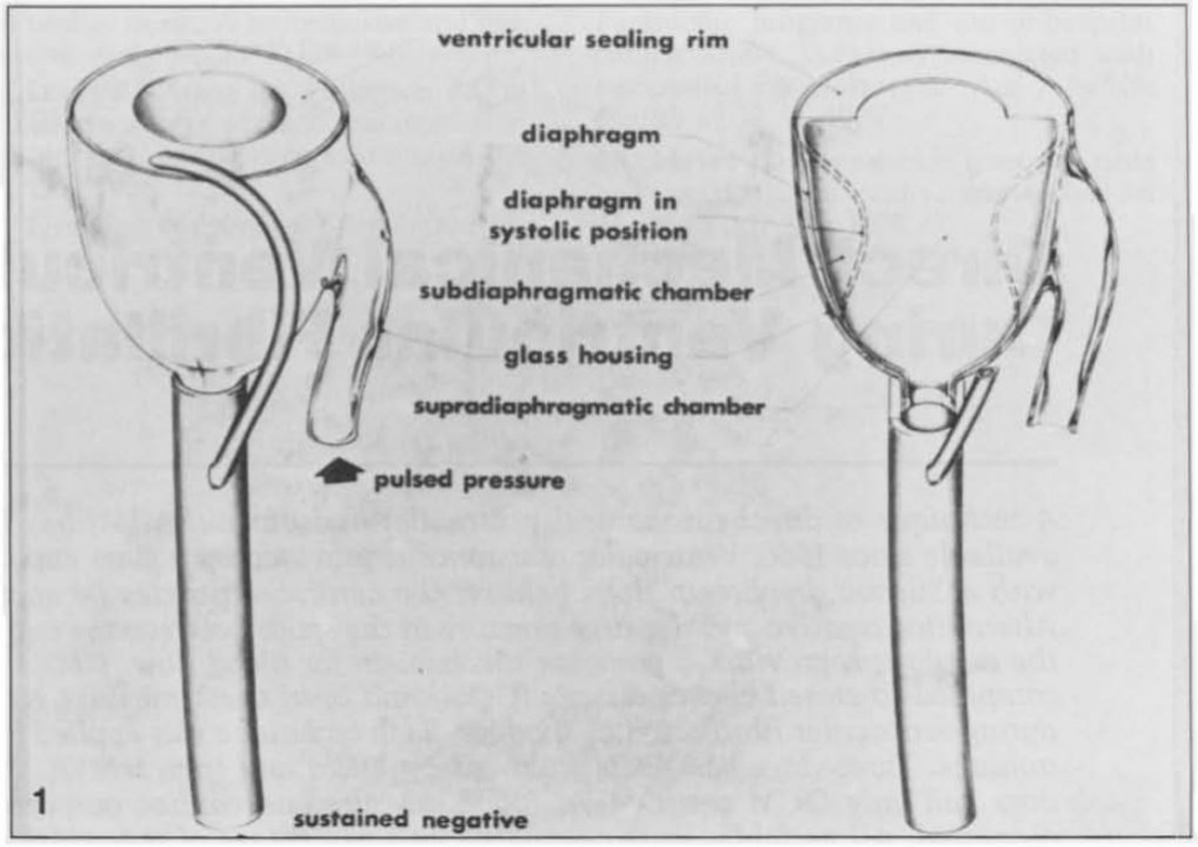
**The “Anstadt Cup” device for direct mechanical ventricular actuation**. The glass assistor cup was held in place by suction [26]. Reproduced with permission from [27] McCabe JB, Ventriglia WJ, Anstadt GL, Nolan DJ. Direct mechanical ventricular assistance during ventricular fibrillation. *Ann Emerg Med*. 1983; 12: 739–44.

**Direct mechanical ventricular actuation** was initially described in 1966 by Anstadt, Schiff, and Baue. In this technique, a glass assistor cup with a flexible diaphragm (known as the Anstadt Cup) was held onto the heart by suction. Cardiac output was generated by direct mechanical compression of the ventricles (Fig. 2, Ref. [26, 27]) [26]. The Anstadt Cup was able to support the circulation for 2–3 days in animals with ventricular fibrillation; however, long-term experimental results were never satisfactory [28]. In humans, the device was tested in only a few clinical trials, in which it was applied after prolonged periods of open cardiac massage ranging from 40 minutes to 12 hours [29, 30]. Although hemodynamic improvements were noted in these desperate cases, no clinical trial was ultimately successful [29, 31, 32].

**Fig. 3.**
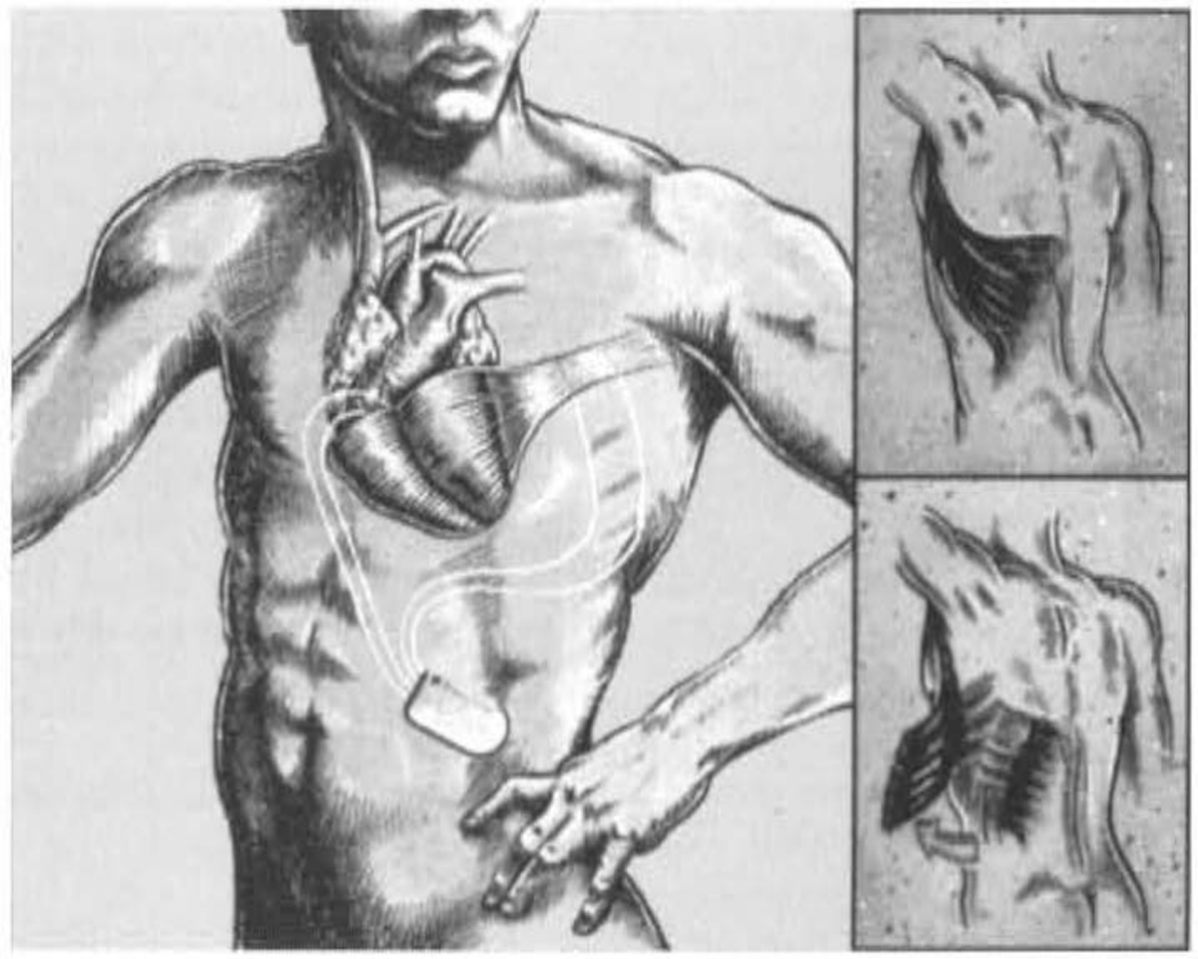
**Diagram of dynamic cardiomyoplasty with latissimus dorsi wrapped around both ventricles**. Reproduced with permission from [34] Letsou GV, Austin L, Grandjean PA, Braxton JH, Elefteriades JA. Dynamic cardiomyoplasty. Cardiology Clinics. 1995; 13: 121–124.

**Dynamic cardiomyoplasty** mimicked the effects of mechanical ventricular assistance by using autologous muscle to surround and contract the heart (Fig. 3) [33, 34]. In this approach, the patient’s latissimus dorsi was first electrically stimulated and “trained” to become fatigue resistant by using a pacemaker capable of producing the train of electrical impulses necessary for skeletal muscle contraction. In a subsequent operation, the latissimus dorsi was disconnected from its insertion, translocated to encircle the heart, and then stimulated to contract in synchrony with it [33]. In experimental animal models of dynamic cardiomyoplasty, the expected alterations in systemic arterial pressure were produced [34]. In a prospective randomized study in humans, dynamic cardiomyoplasty improved New York Heart Association (NYHA) class in the experimental arm but had not objectively improved cardiac performance parameters at 12 months or conferred a survival advantage when compared with controls [35]. Dynamic cardiomyoplasty did prevent progressive left ventricular dilation, which may have accounted for the overall improvements in NYHA classification and quality of life. The latissimus dorsi’s “girdling” effect during diastole was postulated to be as important as active muscle contraction during systole.

**Fig. 4.**
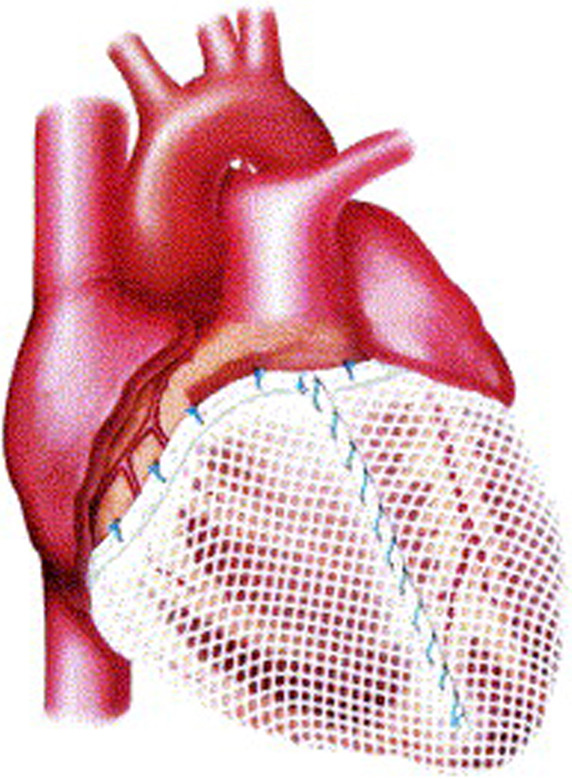
** Acorn CorCap cardiac support device in place, covering both ventricles**. Reproduced with permission from [36] Sabbah HN. The Cardiac Support Device and the Myosplint: treating heart failure by targeting left ventricular size and shape. Annals of Thoracic Surgery. 2003; 75: S13–19.

Other prosthetic devices were developed to reproduce the girdling effect seen in dynamic cardiomyoplasty. The **Acorn CorCap** cardiac support device (Acorn Cardiovascular, St Paul, MN, USA) was a passive-support synthetic mesh wrap that encircled the right and left ventricles. Constructed of preformed polyester polymer, the CorCap was inserted via a thoracotomy and was anchored to the atrioventricular groove with stay sutures to fit snugly about the heart (Fig. 4) [36]. In animals with induced left ventricular dilation and heart failure, application of the CorCap device improved hemodynamics and overall cardiac function, including ejection fraction [37, 38]. In a human clinical trial, improvements in left ventricular end-diastolic volume, end-systolic volume, and sphericity were observed, along with better Living with Heart Failure scores [39]. Nonetheless, the device was difficult to apply and did not progress beyond its initial clinical trial.

**Fig. 5.**
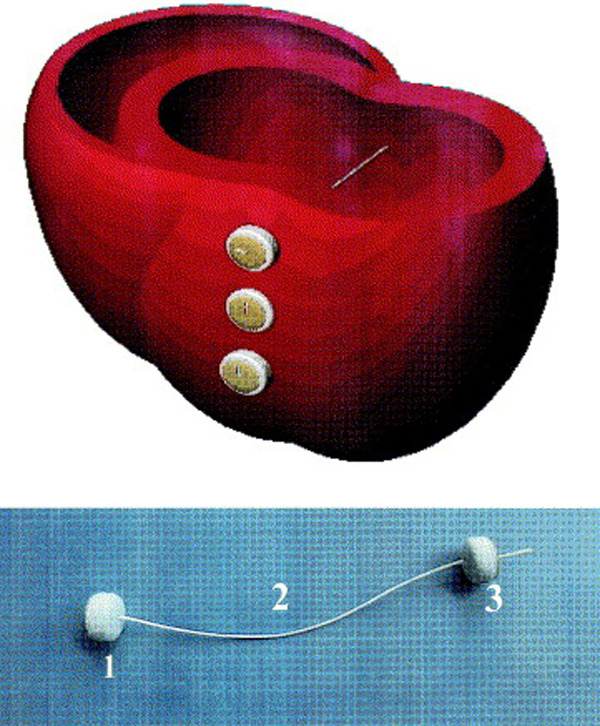
**Cross-section of cardiac ventricles showing the positioning of the Myosplint device**. (Top) Three deployed transventricular splints that bisect the left ventricle. (Bottom) Myosplint components: fixed pad (1), tension member (2), and deployable pad (3). Reproduced with permission from [36] Sabbah HN. The Cardiac Support Device and the Myosplint: treating heart failure by targeting left ventricular size and shape. Annals of Thoracic Surgery. 2003; 75: S13–19.

The **Myosplint** (Myocor, Maple Grove, MN, USA) was another passive-support device designed to decrease the wall stress produced by significant, progressive left ventricular dilation in patients with late-stage heart failure. The device comprised three sets of transventricular filaments connected to a pair of epicardial pads placed on the anterior and posterior aspects of the heart overlying the septum (Fig. 5) [36, 40]. A sternotomy was required for placement. The three sets of pads and transventricular filaments were placed down the left ventricular long axis and were adjusted to draw the opposing ventricular walls together. The Myosplint was effective in animal models of heart failure, significantly reducing left ventricular end-systolic volume and end-systolic wall stress acutely and at 1 month [40]. Mitral valve function did not change, and no significant mitral regurgitation was induced, nor was bleeding at the epicardial pad sites a significant problem. In Europe, Myosplints were implanted successfully in a clinical trial without significant complications or early adverse events. However, objective and subjective improvements in cardiac function were difficult to document. The device is not being used currently [41].

Other devices that provide cardiac support through direct compression are under investigation. The **Adjucor **cardiac assist system is a single-layer compression device inserted via thoracotomy or sternotomy that provides direct cardiac compression in synchrony with the cardiac cycle [42]. It is currently in animal trials. The C-Pulse cardiac assist system provides mechanical counterpulsation to the ascending aorta [43]. Further efforts at developing this device have apparently ceased.

## 3. The CorInnova Direct Cardiac Compression Device

An implantable direct cardiac compression device without blood contact that provides diastolic support and girdling is being developed by CorInnova, Inc. This low-profile device is placed within the pericardium and can be inserted in a minimally invasive manner. The device is designed to provide biventricular support, increased cardiac output, and ventricular unloading in patients with heart failure. The CorInnova device may provide an effective device-based solution for heart failure patients who currently have few or limited mechanical cardiac support options, including those with biventricular cardiac failure, those with right heart failure, those who are older, and those who are of smaller size. An important, unique feature of this technology is the provision of mechanical cardiac assistance without blood contact or need for anticoagulation. The CorInnova device may be particularly important for those patients who have contraindications to anticoagulation due to allergy, neurological bleeds, or preexisting hemorrhage. No other mechanical circulatory support device addresses these underserved heart-failure populations.

### 3.1 Device Design and Physiology

**Fig. 6.**
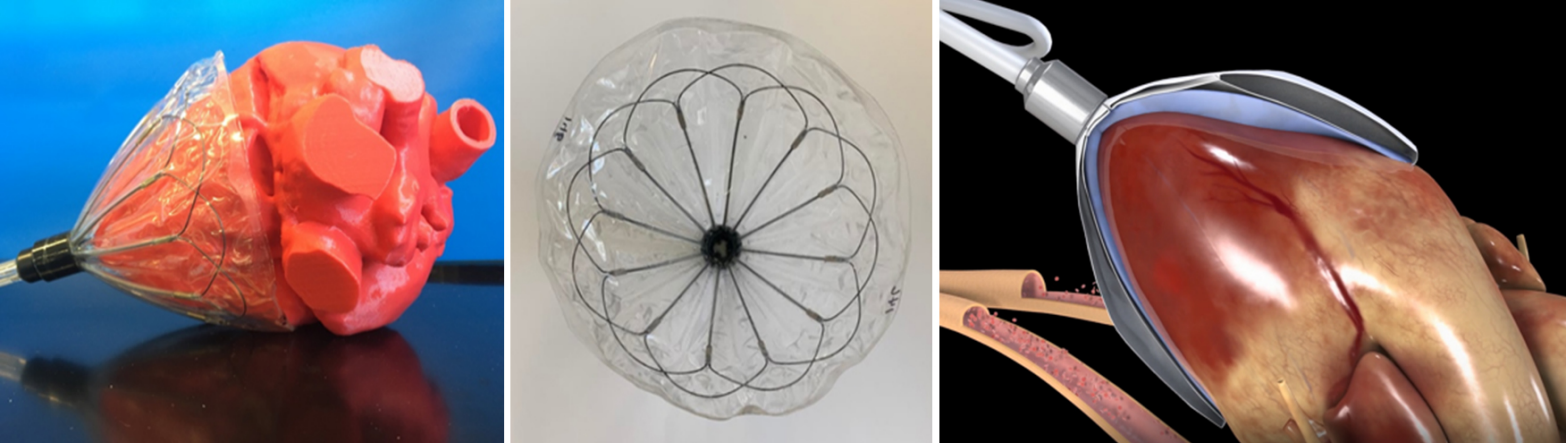
**The CorInnova device**. Left: The cup-shaped device is deployed inside the pericardial sac around the ventricles. Shown is a side view of the device around a 3D-printed ovine heart. Middle: Top view of device, which consists of a Nitinol frame that allows self-deployment. Right: The device includes an inner (epicardial) fluid-filled polyurethane film buffering component and an outer polyurethane film active assist component [44, 45]. Images courtesy of CorInnova, Inc.

The CorInnova device is situated within the pericardium and surrounds both ventricles. The device consists of two concentric sets of thin-film polyurethane chambers: (1) an inner (epicardial) set of saline-filled chambers that conform intimately to the epicardial surface, eradicating any gaps in the interface between the device and heart; and (2) an outer set of air-filled chambers cycled to inflate and deflate in synchrony with the heart. The outer air-filled chambers provide active epicardial compression during systole and negative epicardial pressure during diastole, consistent with physiological cardiac contraction and relaxation. A superelastic Nitinol frame gives the device structure, enables minimally invasive self-deployment, and enhances diastolic filling (Fig. 6, Ref. [44, 45]).

The device is triggered by cardiac electrical activity. The implanted component of the device contains sensing electrodes to acquire a native electrocardiogram (ECG). The amplitude and resolution of the ECG signal acquired at the heart are significantly more robust than that those acquired from skin-surface leads [36], enhancing triggering accuracy and reliability. The electrodes and driveline are routed together externally through the device’s driveline, where they are connected to the external controller and closed-volume pneumatic drive system. The device comprises an external controller and a pneumatic driver that applies vacuum and pressure. Systemic blood pressure is monitored but does not influence device timing or compression. Whereas the pneumatic drive system is conceptually similar to that used in devices such as the IABP, the CorInnova controller’s settings are highly customizable and allow the operator to refine the timing and volume of gas delivered to the device, as well as to control inflation and deflation sequencing of the individual isolator disks for optimal physiological ventricular compression and relaxation assist. The isolator disks serve as a fail-safe that pneumatically decouples the system source pressure and vacuum from the implanted component by limiting the total potential volume that can be delivered to the device. This mitigates potential overpressurization. A custom cardiac trigger monitor and custom driver software allow for reliable ECG acquisition and R-wave triggering, as established in almost 30 large-animal studies. In instances of cardiac arrhythmia, the system is designed simply to refrain from assistance. The system permits 1:1, 1:2, and 1:3 assists, similar to an IABP, allowing the device to function appropriately at heart rates higher than 110 beats per minute.

**Fig. 7.**
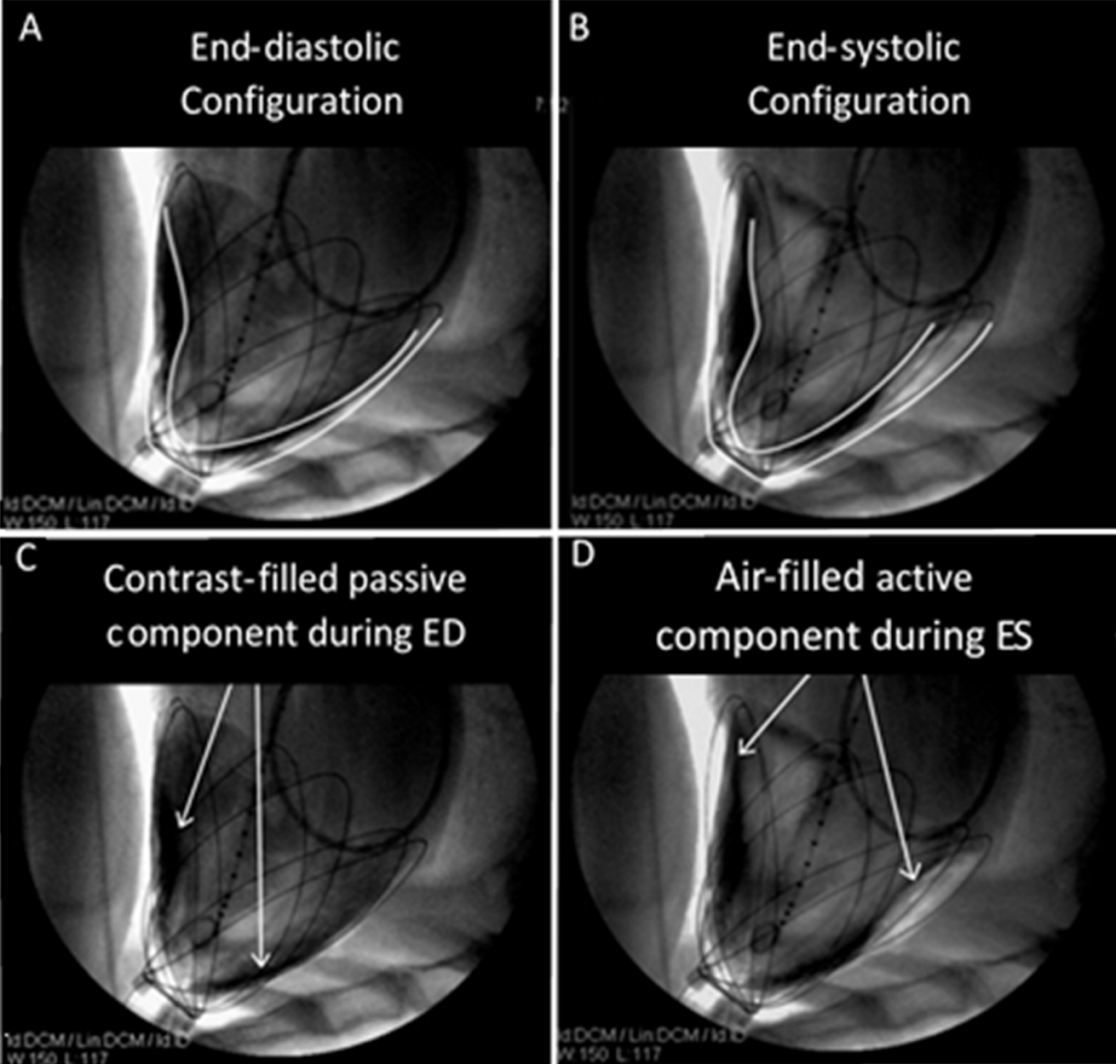
**Fluoroscopic imaging and contour tracings of the heart surface and CorInnova device in acute animal studies**. Unlike previous epicardial assist devices, the CorInnova device assists without inverting native heart curvature (A,B). The device becomes intrinsically pneumatically coupled to the heart as soon as the fluid component is filled (C) and free air is evacuated from the chest, so that the heart is not ejected from the device during assist (D). This allows the device to be implanted without invasive attachment methods, such as suturing or vacuum). Reproduced with permission from [47] Moreno MR, Biswas S, Harrison LD, Pernelle G, Miller MW, Fossum TW, Nelson DA, Criscione JC. Assessment of Minimally Invasive Device that Provides Simultaneous Adjustable Cardiac Support and Active Synchronous Assist in an Acute Heart Failure Model. The Journal of Medical Devices. 2011; 5(4): 041008.

Previous devices, such as the Anstadt Cup, featured a rigid frame and aggressive compression that inverted the normal curvature of the ventricles, leading to bruising and damage of the heart tissue. Conversely, the CorInnova device is constructed on a more compliant Nitinol frame [44, 46]. Cardiac bruising has not been identified in experimental studies using the CorInnova device (Fig. 7, Ref [47]). Anatomical coronary artery specimens from animals after 5–7 days of cardiac assist with the CorInnova device have shown no coronary artery damage [48].

The CorInnova device has multiple advantages over previous devices. The device’s chambers are fabricated from soft polyurethane and are uniquely designed to couple to the heart. The outer air-filled chambers and inner saline-filled chambers work together in such a way that radial expansion of the device uniformly applies low pressure (15–20 mmHg) to the heart in synchrony with the native cardiac contraction. Cardiac output and mean arterial pressure are augmented without inversion of the cardiac ventricles’ curvature; the device unloads both ventricles and promotes appropriate cardiac motion [48]. Although passive intrapericardial constraint devices exist (Acorn CorCap, Mardil Medical) [49], no other implantable extracardiac active assist device allows for minimally invasive placement and activation within the pericardial space.

**Fig. 8.**
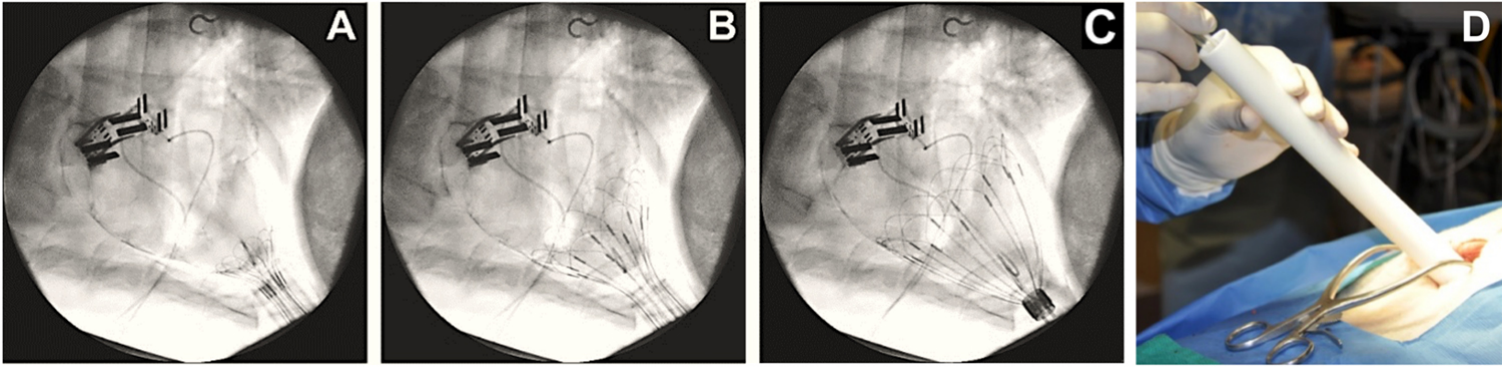
**CorInnova device delivery**. (A–C) Fluoroscopic images showing use of the deployment tube and self-deploying wire frame to successfully place the device in the pericardial sac. (D) The surgeon pushes the device out of the delivery tube and into the pericardial sac. Deployment with this method has a success rate of 100% to date and an average placement time of <20 seconds once the tube is placed at the pericardial opening. Fig. 8A–C, adapted from [44] Hord EC, Bolch CM, Tuzun E, Cohn WE, Leschinsky B, Criscione JC. Evaluation of the CorInnova heart assist device in an acute heart failure model. The Journal of Cardiovascular Translational Research. 2019; 12: 155–163, under Creative Commons License CC BY 4.0 (http://creativecommons.org/licenses/by/4.0/). Fig. 8D courtesy of CorInnova, Inc.

Minimally invasive placement is an important and underappreciated advantage of the CorInnova device, which in animals is inserted through a small subxiphoid incision and opening at the apex of the pericardium (Fig. 8, Ref. [44]). In humans, the approach being developed begins with a minithoracotomy over the cardiac point of maximal impulse, followed by a completely catheter-based insertion guided by echocardiographic visualization of the pericardial sac. Minimally invasive placement allows the pericardial sac to remain intact. An intact pericardium stabilizes the device in an appropriate position without the need for cardiac anchoring sutures, and it also should prevent the heart from being pushed out of the device when the device compresses the heart [48]. This eliminates the anchoring challenges observed with the Anstadt Cup, Acorn CorCap, and dynamic cardiomyoplasty [50].

A vital advantage of the CorInnova device is its non–blood-contacting mode of mechanical cardiac assist. All current temporary mechanical cardiac assist devices—IABPs, extracorporeal membrane oxygenation (ECMO), CentriMag (Abbott, Abbott Park, IL, USA), TandemHeart (LivaNova, London, UK), and Impella (Abiomed, Danvers, MA, USA)—are placed within the vascular system and therefore share a common set of significant adverse events associated with blood contact, such as bleeding, thrombosis, vascular insult, and neurological injury [51, 52]. In contrast, epicardial compression with the CorInnova device does not require vascular contact. An additional important benefit of this lack of blood contact is that cardiac assist can be reduced or halted as needed or desired. None of the other currently available mechanical cardiac assist devices can be deactivated while in place, due to the potential for device thrombosis or stroke. Because the CorInnova device avoids blood contact, device assistance can be easily and safely reduced in a gradual manner and even suspended (either intermittently or for extended durations) to assess the patient’s native heart function. This feature offers a greater potential to wean patients off cardiac support as their status improves, compared with blood-contacting devices, and allows for extended periods away from the intensive care unit for ambulation and rehabilitation.

The CorInnova device provides pulsatile flow for circulatory support. The importance of pulsatile flow, as opposed to continuous flow, is unclear at present. Whereas most existing mechanical cardiac assist devices provide support by continuous flow, the scientific debate over the benefits of pulsatile flow versus continuous flow is still unsettled [12]. Pulsatile flow is more physiological, and multiple studies have shown that pulsatile flow increases production of nitric oxide (a signaling molecule that has vasodilatory function and increases blood flow) and better preserves end-organ perfusion and function [53]. These effects are more pronounced in high-risk patients and those with preexisting organ dysfunction [54]. Continuous-flow pumps have become the dominant method for providing cardiac support over the last decade. Other than IABPs, the CorInnova device will be the only device to provide physiological pulsatile cardiac assist—and with greater efficacy than IABPs.

Ventricular unloading in patients with heart failure reduces end-diastolic wall stress [55], which when elevated is a well-established pathological driver of maladaptive ventricular remodeling that can lead to worsening or de novo heart failure. Ventricular unloading through decreased filling pressures and a reduction in the heart’s contribution to total left ventricular stroke work improves cardiac function in patients with heart failure. In pilot animal studies [45], the CorInnova device unloaded the ventricle, reducing left ventricular end diastolic pressure and the proportion of total left ventricular stroke work contributed by the heart (left ventricular volume was not assessed), while increasing cardiac output in animal models of cardiogenic shock and heart failure. This is accomplished not only through hemodynamic support (comparable to the Impella), but also through reducing left ventricular wall tension by directly contacting and mechanically supporting the myocardium from the epicardial surface.

The less-invasive surgical implantation of the CorInnova device and its easy positioning within the pericardial sac are ideal for fragile patients with heart failure who may be awaiting heart transplant. The device does not require suturing for appropriate positioning or cardiac chamber cannulation, either of which could complicate subsequent transplant. In animal trials, the CorInnova device was quickly and easily explanted after 10 days, without damage to the myocardium [48].

### 3.2 Potential and Practical Impact

The CorInnova device is intended as a heart failure treatment in patients requiring mechanical cardiac support primarily in elective or semi-elective situations. It is uniquely suited for patients with biventricular failure, for patients with contraindications to anticoagulation, for smaller-sized patients, for older patients, for patients who may benefit from intermittent cardiac support to allow for rehabilitation before other procedures (including transplant), and for patients who are not good candidates for current blood-contacting intravascular cardiac assist devices.

Preimplantation preparations are anticipated to include the typical care for any patient admitted with severe heart failure, including an echocardiogram to help with choosing an appropriately sized CorInnova device. Six device sizes are being tested in current animal experiments, whereas eight sizes will be available for humans. In animals, appropriate device size is determined after anesthesia is induced by using intraoperative fluoroscopy in conjunction with a custom sizing tool and directly measuring the distance from the cardiac apex to the atrioventricular groove anteriorly and posteriorly, which allows calculation of the heart’s diameter at the atrioventricular groove. In humans, it is anticipated that appropriate sizing will be determined preoperatively using echocardiography or cardiac magnetic resonance imaging and confirmed intraoperatively using the same direct measurement of the distance from apex to atrioventricular groove that has been used successfully in animals.

Bridge to recovery has become more popular in mechanical cardiac assist, a strategy for which the CorInnova device is ideally suited [56]. The device’s ability to provide biventricular support reduces the risk for right heart failure, which occurs in 20%–30% of patients with isolated left ventricular cardiac assistance [57]. The avoidance of anticoagulation adds further to the potential attractiveness of the CorInnova device for bridge to recovery.

The CorInnova device may be ideal not only for short-term use in humans (for which approval is being sought), but also for intermediate-term use (months to 1 year), given that it can be used intermittently, does not require anticoagulation, and does not require emergency attention if it malfunctions. More than 55% of long-term complications related to ventricular assist devices arise from the blood-device interface [58]. These thrombotic and bleeding complications should not occur with the CorInnova device. Long-term use of the CorInnova device offers the opportunity to better understand cardiac recovery, myocardial reverse remodeling, and the long-term effects of heart failure on other organ systems, as the device is much more likely than currently available devices to be tolerated for months or even years. The CorInnova device may more effectively induce cardiac recovery by correcting heart motion and reducing left ventricular wall stress—key factors in restorative cardiac reverse remodeling.

Given that the CorInnova device can be easily scaled to fit a wide range of heart sizes, it may be of special benefit to pediatric patients. Scaling ability has been shown in two unpublished pilot pediatric animal studies. The low-profile, extracardiac design of the device requires less intrathoracic space than do fully implantable pumps, and the system has only one driveline—ideal features for small patients who are currently limited to highly-invasive extracardiac devices such as ECMO, the most commonly used cardiac assist method for pediatric patients, or the EXCOR Pediatric device (Berlin Heart, Berlin, Germany). A new therapy that reduces adverse event rates is urgently needed.

Expected possible relative contraindications for the CorInnova device include pericardial adhesions, previous cardiac surgery, unpredictable or intractable arrhythmias, lack of pericardial integrity, and cardiac geometry that is outside of the range of offered device sizes. Large pericardial effusions may lead to device instability if the pericardium has enlarged sufficiently, but tightening the pericardium by using a purse-string suture at or near the pericardiotomy might address this potential difficulty. Devices placed within the heart, such as automatic implantable cardioverter defibrillators, pacemakers, and cardiac resynchronization devices, are not expected to contraindicate implantation or to affect device function.

## 4. Conclusions

Cardiac compression for resuscitation has been an intriguing subject for medical investigations for hundreds of years. The CorInnova cardiac assist device is an extension of these efforts. It is a thin-film polyurethane cardiac compression device mounted on a collapsible Nitinol wire frame that allows for easy deployment within an intact pericardium. Although it appears superficially similar to previous direct-compression devices, such as the Anstadt Cup, the CorInnova device is dramatically different, providing both active and passive cardiac support. Preclinical testing has been extremely promising, with improvements in cardiac output and other cardiac parameters in animal heart failure models. The CorInnova device does not require anticoagulation, can be removed easily, and requires minimal maintenance compared with other cardiac assist devices. It is a potentially transformative technology that is moving rapidly toward first-in-human use.
